# Dynamic disease‐associated gene regulatory transitions unmask genetic risk associated neurodegenerative cell states

**DOI:** 10.1002/alz.088018

**Published:** 2025-01-03

**Authors:** Xia Han, Jessica E Rexach

**Affiliations:** ^1^ UCLA, Los Angeles, CA USA; ^2^ Lawrence Chen Program in Neurogenetics, Department of Neurology, David Geffen School of Medicine, University of California, Los Angeles, CA USA

## Abstract

**Background:**

Abnormal tau protein accumulation selectively affects distinct brain regions and specific neuron and glia populations in tau‐related dementias like Alzheimer’s disease (AD), frontotemporal dementia (FTD, Pick’s disease type), and Progressive supranuclear palsy (PSP). The regulatory mechanisms governing cell‐type vulnerability remain unclear.

**Method:**

In a cross‐disorder single‐nucleus analysis, we examined 663,896 nuclei, assessing chromatin accessibility in three brain regions (motor cortex, visual cortex and insular cortex) across PSP, AD, and FTD in 40 individuals. Integrating genetic data with single‐nucleus RNA sequencing, we identified cell‐type‐specific cis‐regulatory elements (CREs) influencing disease‐associated transcriptional changes. Connecting GWAS signals to cell types enabled precise mapping of risk‐associated genetic elements to disorder‐specific gene regulatory events, accounting for dynamic gene regulation in the diseased brain.

**Result:**

Disorder enriched CRE modules were primarily activated in PSP astrocytes and FTD microglia. PSP exhibited increased astrocyte involvement, identified by distinct changes in astrocytes (ast.C1). In contrast, FTD featured microglia with suppressed GRN expression (mg.C4). Differentially accessible gene regulatory elements in mg.C4 uniquely and significantly contributed to FTD risk heritability across microglia. Disease dynamic chromatin accessibility peaks and gene enhancer elements, especially in astrocytes, accounted for more AD and PSP heritability, while FTD heritability predominantly involved microglial upregulated peaks.

**Conclusion:**

This study enhances our understanding of disease heritability, reinforcing the multicellular model of neurodegeneration. Glial cell types interacting with disease‐specific elements form disorder‐specific networks, deepening knowledge of genomic and cellular mechanisms in tau‐associated neurodegeneration.

